# A non-canonical role for the proneural gene *Neurog1* as a negative regulator of neocortical neurogenesis

**DOI:** 10.1242/dev.157719

**Published:** 2018-10-01

**Authors:** Sisu Han, Daniel J. Dennis, Anjali Balakrishnan, Rajiv Dixit, Olivier Britz, Dawn Zinyk, Yacine Touahri, Thomas Olender, Marjorie Brand, François Guillemot, Deborah Kurrasch, Carol Schuurmans

**Affiliations:** 1 Biological Sciences Platform, Sunnybrook Research Institute, Toronto, ON M4N 3M5, Canada; 2 Department of Biochemistry, University of Toronto, Toronto, ON M5S 1A8, Canada; 3 Department of Biochemistry and Molecular Biology, Alberta Children's Hospital Research Institute, Hotchkiss Brain Institute, University of Calgary, Calgary, AB T2N 4N1, Canada; 4 Department of Molecular Genetics, Alberta Children's Hospital Research Institute, University of Calgary, Calgary, AB T2N 4N1, Canada; 5 The Francis Crick Institute-Mill Hill Laboratory, London NW7 1AA, UK; 6 Ottawa Hospital Research Institute, Ottawa, ON K1H 8L6, Canada

**Keywords:** Proneural genes, bHLH transcription factors, Neurogenins, Neocortex, Neurogenesis

## Abstract

Neural progenitors undergo temporal identity transitions to sequentially generate the neuronal and glial cells that make up the mature brain. Proneural genes have well-characterised roles in promoting neural cell differentiation and subtype specification, but they also regulate the timing of identity transitions through poorly understood mechanisms. Here, we investigated how the highly related proneural genes *Neurog1* and *Neurog2* interact to control the timing of neocortical neurogenesis. We found that *Neurog1* acts in an atypical fashion as it is required to suppress rather than promote neuronal differentiation in early corticogenesis. In *Neurog1^−/−^* neocortices, early born neurons differentiate in excess, whereas, *in vitro*, *Neurog1^−/−^* progenitors have a decreased propensity to proliferate and form neurospheres*.* Instead, *Neurog1^−/−^* progenitors preferentially generate neurons, a phenotype restricted to the *Neurog2*^+^ progenitor pool. Mechanistically, Neurog1 and Neurog2 heterodimerise, and while *Neurog1* and *Neurog2* individually promote neurogenesis, misexpression together blocks this effect. Finally, *Neurog1* is also required to induce the expression of neurogenic factors (*Dll1* and *Hes5*) and to repress the expression of neuronal differentiation genes (*Fezf2* and *Neurod6*). *Neurog1* thus employs different mechanisms to temper the pace of early neocortical neurogenesis.

## Abstract (precis)

**Summary:** Proneural genes are well known inducers of neurogenesis; however, *Neurog1* induces the expression of Notch pathway genes and represses the expression of neuronal differentiation genes.

## INTRODUCTION

Time is an important axis of developmental information in the neocortex; progenitor cells undergo precise temporal identity transitions that define the numbers and types of neuronal and glial cells that are born at any given time (Pearson and Doe, 2004). The first cortical cells to be born are excitatory pyramidal neurons, which form six layers in a sequential inside-out manner between embryonic day (E) 10.5 and E17 in mouse, with deep layers born first and outer layers last (Takahashi et al., 1995). At the end of the neurogenic period, cortical progenitors become gliogenic, giving rise to astrocytes in late embryogenesis and to oligodendrocytes in the early postnatal period (Kessaris et al., 2006; Piper et al., 2010; Subramanian et al., 2011).

Temporal identities are encoded at the progenitor cell level in the neocortex (Pearson and Doe, 2004). Cortical progenitors include radial glial cells (RGCs), the cell bodies of which lie in the ventricular zone (VZ) of the dorsal telencephalon (reviewed by Kriegstein and Alvarez-Buylla, 2009). RGCs either divide symmetrically to form additional RGCs, expanding the progenitor pool, or asymmetrically to generate another RGC (to self-renew) and either a neuron or intermediate neuronal progenitor (INP). INPs are a secondary pool of cortical progenitors that lose their ventricular contacts and form a subventricular zone (SVZ) (Haubensak et al., 2004; Miyata et al., 2004; Noctor et al., 2004). INPs divide once or twice before differentiating into neurons that populate all six neuronal layers (Kowalczyk et al., 2009), passing positional information onto their neuronal progeny (Elsen et al., 2013).

Intrinsic cell determinants confer temporal cortical identities (Pearson and Doe, 2004). Included are the proneural genes, which encode basic-helix-loop-helix (bHLH) transcription factors that promote neurogenesis, specify subtype identities and control the timing of cortical progenitor cell identity transitions (Bertrand et al., 2002; Wilkinson et al., 2013). Indeed, upper-layer neurons (Dennis et al., 2017) and astrocytes (Nieto et al., 2001) are generated prematurely in *Neurog2*^−/−^; *Ascl1*^−/−^ mutants, as are astrocytes in the *Neurod4*^−/−^; *Ascl1*^−/−^ midbrain (Tomita et al., 2000). Currently, it is not well understood how the proneural genes regulate developmental timing.

Three proneural genes are expressed in the cortical VZ; *Neurog1*, *Neurog2* and *Ascl1* (Britz et al., 2006). We focus here on *Neurog1* and *Neurog2*, which have similar expression profiles in the embryonic neocortex (Fode et al., 2000; Gradwohl et al., 1996). *Neurog1* and *Neurog2* also share expression domains and have partially overlapping functions in other CNS regions, including the olfactory bulb (Cau et al., 2002; Shaker et al., 2012), cerebellum (Zordan et al., 2008) and ventral neural tube (Quiñones et al., 2010). In contrast, *Neurog1* and *Neurog2* are expressed in a distinct manner in the peripheral nervous system (PNS), including in the epibranchial placodes, and olfactory epithelium, reflecting a functional divergence (Fode et al., 1998; Ma et al., 1998; Shaker et al., 2012).

In the neocortex, *Neurog2* and *Neurog1* have overlapping and distinct functions. *Neurog2* specifies the glutamatergic identity of early born, deep-layer neurons (Fode et al., 2000; Schuurmans et al., 2004). Hence, in *Neurog2*-null mutants, deep-layer neurons lose their excitatory glutamatergic phenotype and instead acquire an inhibitory GABAergic interneuron fate (Fode et al., 2000; Schuurmans et al., 2004). *Neurog2* is instructive for a glutamatergic neuronal identity, which it confers even outside of its normal expression domain, in the ventral telencephalon (Mattar et al., 2008). Even when overexpressed in early cortical progenitors, *Neurog2* induces the premature differentiation of glutamatergic neurons with phenotypic features of deep layer VI (Tbr1^+^) and V (Ctip2^+^) neurons (Dennis et al., 2017).

In contrast*,* the analysis of *Neurog1* function in neocortical development has led to some paradoxical findings. In *Neurog1*^−/−^; *Neurog2*^−/−^ double mutants, the misspecification of cortical neurons to a GABAergic interneuron identity extends into the caudolateral cortex (Schuurmans et al., 2004), whereas the *Neurog2^−/−^* phenotype is confined to dorsomedial domains. Notably, *Neurog1* expression is lost in *Neurog2*^−/−^ dorsomedial cortical progenitors, such that *Neurog2*^−/−^ and *Neurog1/2^−/−^* cortices are equivalent in this region (Fode et al., 2000; Mattar et al., 2004). *Neurog1* and *Neurog2* are thus functionally redundant for specifying a correct glutamatergic neuronal identity during early corticogenesis. However, in *Neurog1*^−/−^ single mutants, the preplate layer is thicker (Schuurmans et al., 2004), including an expansion of the earliest born Cajal-Retzius neurons (Dixit et al., 2014). Thus, although *Neurog1* and *Neurog2* may have redundancy in their abilities to specify a glutamatergic neuron identity, *Neurog1* also exhibits some properties of a negative regulator of neurogenesis*.* We set out to determine how *Neurog1* inhibits early cortical neurogenesis, revealing cross-inhibitory interactions with *Neurog2*, and revealing that *Neurog1* is required to induce the expression of Notch pathway genes (*Dll1* and *Hes5*) and to repress the expression of neuronal differentiation genes (*Fezf2* and *Neurod6*).

## RESULTS

### *Neurog1* and *Neurog2* have overlapping yet temporally distinct expression profiles in the developing neocortex

**Fig. 1.**
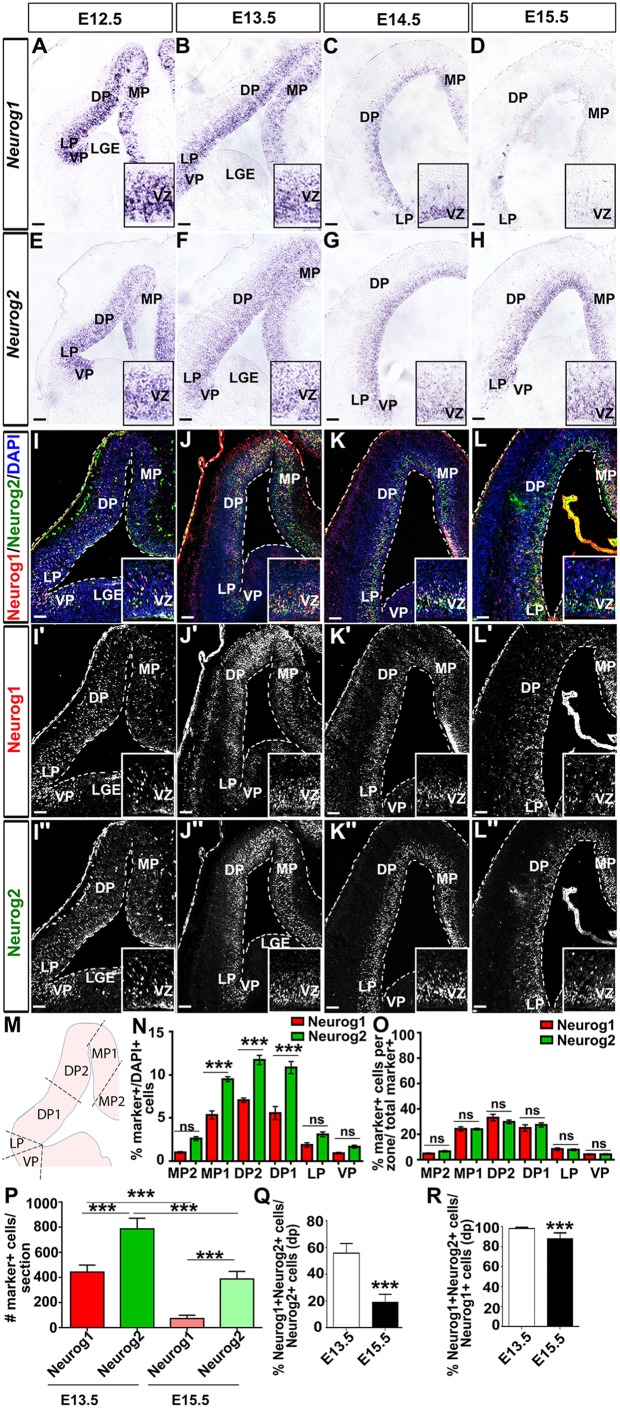
**Temporal analysis of *Neurog1* and *Neurog2* expression in the developing neocortex.** (A-H) Distribution of *Neurog1* (A-D) and *Neurog2* (E-H) transcripts at E12.5 (A,E), E13.5 (B,F), E14.5 (C,G) and E15.5 (D,H). (I-L″) Co-expression of Neurog1 and Neurog2 (I-L), Neurog1 (I′-L′), and Neurog2 (I″-L″) protein at E12.5 (I-I″), E13.5 (J-J″), E14.5 (K-K″) and E15.5 (L-L″). Insets show high magnification images of dorsal pallium. 2.5× magnifications (A-H) and 2× magnifications (I-L″). (M) Schematic of pallial zones of the dorsal telencephalon. (N) Quantification of the percentage of Neurog1- and Neurog2-expressing DAPI^+^ nuclei per pallial zone (*N*=3, *n*=9; one-way ANOVA with post-hoc Tukey). (O) Quantification of the proportional distribution of Neurog1 and Neurog2 per pallial zone (*N*=3, *n*=9; one-way ANOVA with post-hoc Tukey). (P) Quantification of the number of Neurog1^+^ and Neurog2^+^ progenitors per cortical field at E13.5 and E15.5 (*N*=3, *n*=9; one-way ANOVA with post-hoc Tukey). (Q) The percentage of the Neurog2^+^ progenitor pool that co-expresses Neurog1 at E13.5 and E15.5 (*N*=3, *n*=9; two-tailed *t-*test). (R) The percentage of the Neurog1^+^ progenitor pool that co-expresses Neurog2 at E13.5 and E15.5 (*N*=3, *n*=9; two-tailed *t-*test). Data are mean±s.e.m. ***P<0.001. MP, medial pallium; DP, dorsal pallium; LP, lateral pallium; VP, ventral pallium, LGE, lateral ganglionic eminence. Scale bars: 100 µm in A-H (40 µm in insets); 75 µm in I-L″ (32.5 µm in insets).

*Neurog1* and *Neurog2* are both expressed in dorsal telencephalic (pallial) progenitors (Britz et al., 2006; Fode et al., 2000). To test whether their apparent diverse functions could be due to temporal or spatial differences, we performed a detailed comparison of their expression patterns at the transcript and protein level between E12.5 and E15.5. At E12.5, both *Neurog1* (Fig. 1A) and *Neurog2* (Fig. 1E) mRNA were detected throughout the dorsal telencephalic VZ, including in the medial, dorsal and lateral pallium, and rounding the corticostriatal angle into the ventral pallium (Fig. 1A,M; pallial domains defined as described by Yun et al., 2001). Transcript distribution was graded, accumulating more densely in ventrolateral most domains for both *Neurog1* (Fig. 1A) and *Neurog2* (Fig. 1E). A very similar pattern of expression was seen at E13.5 and E14.5, with robust expression of both *Neurog1* (Fig. 1B,C) and *Neurog2* (Fig. 1F,G) throughout the pallial VZ. However, by E15.5, *Neurog1* transcripts were detected at low levels in the pallium (Fig. 1D), whereas *Neurog2* continued to be highly expressed (Fig. 1H).

We next asked whether protein expression matched the *Neurog1* and *Neurog2* transcript distribution. At E12.5 (Fig. 1I-I″; Fig. S1A-D), E13.5 (Fig. 1J-J″; Fig. S1E-H) and E14.5 (Fig. 1K-K″; Fig. S1I-L), both Neurog1 and Neurog2 protein were detected throughout the pallial VZ in scattered progenitor cells in a characteristic ‘salt-and-pepper’ pattern, showing a high ventrolateral-to-low medial gradient. Quantitation of Neurog1^+^ and Neurog2^+^ cortical progenitors in the different pallial territories at E13.5 (Fig. 1M) revealed that there were more Neurog2^+^ versus Neurog1^+^ progenitors in most domains (medial pallium 1, dorsal pallium 1 and 2; Fig. 1N). However, when comparing the proportion of the total Neurog1^+^ and Neurog2^+^ pool in each domain, the overall distribution of these two proteins was very similar (Fig. 1O), even though fewer cortical progenitors expressed Neurog1.

By E15.5, Neurog1 protein was detectable in even fewer cells than Neurog2 (Fig. 1L-L″; Fig. S1M-P); at E13.5 there were 1.77-fold more Neurog2^+^ cortical progenitors compared with Neurog1^+^ cells, whereas at E15.5 there were 6.25-fold more Neurog2^+^ progenitors (Fig. 1P). Consequently, the proportion of Neurog2^+^ progenitors that co-expressed Neurog1 also declined from 55.9±2.4% at E13.5 to 19.20±2.0% at E15.5 (Fig. 1Q). The proportion of Neurog1^+^ progenitors that co-expressed Neurog2 also declined, but these rates of co-expression were much higher, with 98.44±0.37% of Neurog1^+^ progenitors co-expressing Neurog2 at E13.5, and 88.08±1.98% co-expressing at E15.5 (Fig. 1R). Thus, even though Neurog1 is expressed in fewer pallial progenitors, Neurog1 and Neurog2 have very similar expression profiles between E12.5 and E15.5, with Neurog2 expressed in most Neurog1^+^ cells. However, by E15.5, Neurog1 expression is turning off, leaving only Neurog2 expressed in most cortical progenitors.

### *Neurog1^−/−^* radial glial cell progenitors proliferate at a reduced rate

**Fig. 2.**
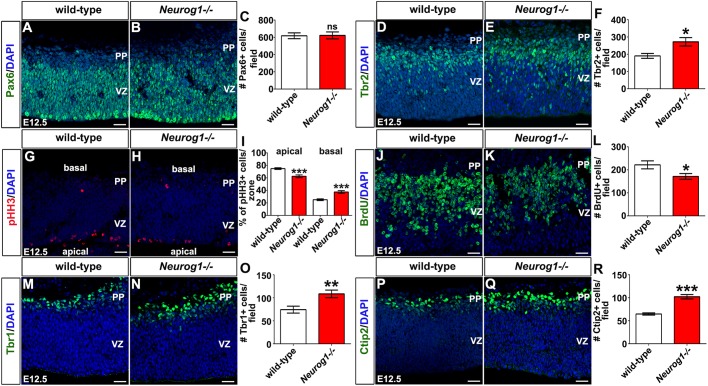
**Characterisation of early *Neurog1^−/−^* cortices.** (A-R) Expression of Pax6 (A,B), Tbr2 (D,E), pHH3 (G,H), BrdU (J,K), Tbr1 (M,N) and Ctip2 (P,Q) in E12.5 wild-type and *Neurog1^−/−^* cortices. Blue is DAPI counterstain. Quantitation of Pax6 (C), Tbr2 (F), pHH3 (I), BrdU (L), Tbr1 (O) and Ctip2 (R) per field of view. *N*=3, *n*=9 for all markers except pHH3, with a two-tailed *t*-test. For pHH3, the entire cortical region was quantitated (*N*=3, *n*=14, two-tailed *t*-test), which was larger than the field of view in G,H. Data are mean±s.e.m. *P<0.05, **P<0.01, ***P<0.001. PP, preplate; VZ, ventricular zone. Scale bars: 25 µm.

To analyse the role of *Neurog1* in cortical development, we first assessed progenitor number in the *Neurog1^−/−^* dorsal pallium (focusing on dp1, defined in Fig. 1M), where both Neurog1 and Neurog2 are expressed at high levels, and at stages between E12.5 and E15.5, during the period when Neurog1 expression first peaks, and then declines. At E12.5, both wild-type and *Neurog1^−/−^* cortices had similar numbers of Pax6^+^ cells, suggesting that the RGC pool was unchanged in the absence of *Neurog1* (Fig. 2A-C). In contrast, the number of Tbr2^+^ INPs was increased in E12.5 *Neurog1^−/−^* cortices compared with wild type (1.43-fold increase; Fig. 2D-F).

Another way to measure apical and basal progenitor pools is to examine the distribution of phospho-histone H3 (pHH3)-positive G2/M-phase cells. Owing to interkinetic nuclear migration, RGCs in G2/M-phase are located at the apical surface, whereas mitotic figures in a basal location are INPs. In *Neurog1^−/−^* cortices there was a decrease to 47.9% in the overall number of pHH3^+^ progenitors (*P*<0.0001; *N*=3, *n*=14; two-tailed *t*-test), primarily affecting the apical pool (Fig. 2G-I). Consequently, the proportion of apical pHH3^+^ cells was reduced to 83.5% in *Neurog1^−/−^* animals, with a corresponding 1.49-fold increase in the proportion of basal pHH3^+^ mitotic figures (Fig. 2G-I). To further assess whether there was a change in the number of dividing cells, we administered a BrdU pulse 30 min prior to dissection to label S-phase progenitors. In E12.5 *Neurog1^−/−^* cortices there was a decrease to 77.3% in the number of BrdU^+^ S-phase progenitors compared to wild type (Fig. 2J-L). Apical RGCs thus proliferate less in *Neurog1^−/−^* cortices, and there is also an increase in Tbr2^+^ INPs, suggesting that the RGC to INP transition is accelerated at E12.5.

### *Neurog1* is required to limit the production of early born cortical neurons

The peak of *Neurog1* expression coincides with the period of layer I and deep-layer neurogenesis. Layer I Cajal-Retzius cells (layer I neurons) are increased in number in E12.5 *Neurog1^−/−^* cortices (Dixit et al., 2014; Fode et al., 2000). Here, we asked whether deep-layer VI and V neurons, which are born from E12.5 onwards, were also altered. We first examined Tbr1, a T-box transcription factor that is expressed in Cajal-Retzius neurons and layer VI corticothalamic neurons (Hevner et al., 2001). There was a 1.46-fold increase in Tbr1^+^ cells in E12.5 *Neurog1^−/−^* cortices compared with wild type (Fig. 2M-O), which could reflect an increase in Cajal-Retzius neurons and/or layer VI neurons. We also examined the expression of Ctip2/Bcl11b, which encodes a zinc-finger transcription factor that is expressed in layer V subcerebral projection neurons (Arlotta et al., 2005; Chen et al., 2008); Ctip2^+^ neurons also increased 1.60-fold in *Neurog1^−/−^* cortices compared with wild type at E12.5 (Fig. 2P-R). Similar results were observed at E13.5 in *Neurog1^−/−^* cortices, with more Tbr1^+^ (1.39-fold increase; Fig. S2A-C) and Ctip2^+^ (1.39-fold increase; Fig. S2D-F) neurons.

To determine definitively whether more deep-layer neurons were born at E12.5 in *Neurog1^−/−^* cortices, we performed birthdating, injecting pregnant dams at E12.5 with BrdU and analysing embryos at E15.5. Although BrdU is diluted out of progenitors that divide, it is retained in post-mitotic neurons derived from S-phase progenitors immediately after they have incorporated the BrdU label. To determine the identity of the newborn postmitotic neurons, cortices of E15.5 wild-type and *Neurog1^−/−^* mice were co-stained with antibodies that recognise Tbr1 and BrdU (Fig. S3A-B′). The number of Tbr1^+^ cells that colocalised with BrdU at E15.5 was 1.24-fold higher in *Neurog1^−/−^* compared with wild-type cortices (Fig. S3C), confirming that E12.5 *Neurog1^−/−^* progenitors have an increased propensity to undergo neurogenesis. The loss of *Neurog1* thus results in a general increase in early born neurons at E12.5, indicating that this gene is a negative regulator of neurogenesis, contrary to its predicted role as a neural determination gene.

### Defects in neurogenesis are rescued by E15.5 in *Neurog1*^−/−^ cortices

We next asked whether the increased production of early-born layer VI and V neurons at E12.5 resulted in a permanent change in these neurons, examining E15.5 cortices (Fig. 1D,L′). The number of Tbr1^+^ (Fig. S3D-F) and Ctip2^+^ (Fig. S3G-I) neurons did not differ significantly from wild-type levels in E15.5 *Neurog1^−/−^* cortices. Thus, the early increase in deep-layer neurogenesis in *Neurog1^−/−^* cortices is compensated for by E15.5. Notably, this compensation is not due to the apoptosis of supernumerary early born neurons, as there was no difference in number of activated caspase 3^+^ cells in E12.5 *Neurog1^−/−^* cortices compared with wild type (Fig. S2G-I).

By E15.5, upper-layer neurons have started to differentiate. Satb2 encodes an AT-rich DNA-binding protein that is expressed in layer II-III callosal neurons (Alcamo et al., 2008; Britanova et al., 2008). At E15.5, the number of Satb2^+^ neurons was not significantly different in *Neurog1^−/−^* compared with wild-type cortices (Fig. S3J-L). There are thus compensatory mechanisms that ensure that the normal complement of cortical neurons is generated in *Neurog1^−/−^* cortices, suggesting that, although *Neurog1* is required to limit early neurogenesis, it is not ultimately essential to control cortical neuronal number.

### Increase in leaving fraction in early embryonic *Neurog1^−/−^* cortices

**Fig. 3.**
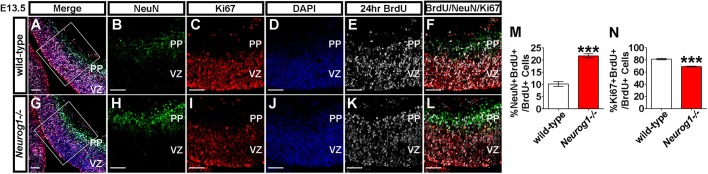
**Bias towards fewer proliferating and more differentiating divisions in E13.5 *Neurog1^−/−^* cortices.** Co-expression of NeuN (green, A,B,F,G,H,L), Ki67 (red, A,C,F,G,I,L) and co-localisation of BrdU (white, A,E,F,G,K,L) and DAPI (blue, A,D,G,J) in E13.5 wild-type (A-F) and *Neurog1^−/−^* (G-L) cortices. (M) Quantitation of the leaving (q-) fraction, which is the proportion of BrdU-incorporating cells that colocalise with NeuN (*N*=3, *n*=9, two-tailed *t*-test). (N) Quantitation of the proliferative (p-) fraction, which is the proportion of BrdU-incorporating cells that colocalise with Ki67 (*N*=3, *n*=9, two-tailed *t*-test). Data are mean±s.e.m. ***P<0.001. PP, preplate; VZ, ventricular zone. The areas outlined in A and G are shown at higher magnification in B-F and H-L, respectively. Scale bars: 50 µm.

As there were more Tbr1^+^ and Ctip2^+^ deep-layer neurons in E12.5-E13.5 *Neurog1^−/−^* cortices, we reasoned that there may be a corresponding shift towards increased differentiation and decreased proliferation by the progenitor pool. To test this assumption, we quantified the leaving (Q) and proliferative (P) fractions at E12.5 and E15.5 by pulse labelling with BrdU 24 h prior to dissection. The Q fraction was calculated by measuring the number of BrdU^+^ cells that expressed the pan-neuronal marker NeuN after 24 h, and the P-fraction was calculated by measuring the number of BrdU^+^ cells that expressed Ki67, a pan-proliferative marker. In E13.5 *Neurog1^−/−^* cortices, the leaving fraction was 2.13-fold higher than in wild-type cortices (Fig. 3A-L,M), consistent with the increased number of early born neurons observed in *Neurog1^−/−^* cortices at early stages. Conversely, we observed a corresponding decrease to 85.0% in the proliferative pool (Ki67^+^BrdU^+^/BrdU^+^) in E12.5 *Neurog1^−/−^* cortices compared with wild type (Fig. 3A-L,N). We also examined the leaving and proliferative fractions at E15.5 (pulse-labelling with BrdU at E14.5), when upper layer neurons are being generated, and when neuronal numbers are similar in wild-type and *Neurog1^−/−^* cortices (Fig. S3D-L). Quantitation of labelled cells revealed that the P fractions (Ki67^+^BrdU^+^/BrdU^+^; Fig. S4A-L,M) and Q fractions (NeuN^+^BrdU^+^/BrdU^+^; Fig. S4A-L,N) were similar in E15.5 wild-type and *Neurog1*^−/−^ cortices. Taken together, these data support the idea that only early stage neocortical progenitors have an enhanced propensity to exit the cell cycle and differentiate precociously in *Neurog1^−/−^* cortices.

### *Neurog1^−/−^* cortical progenitors have a reduced proliferative and self-renewal capacity, and instead preferentially differentiate into neurons

**Fig. 4.**
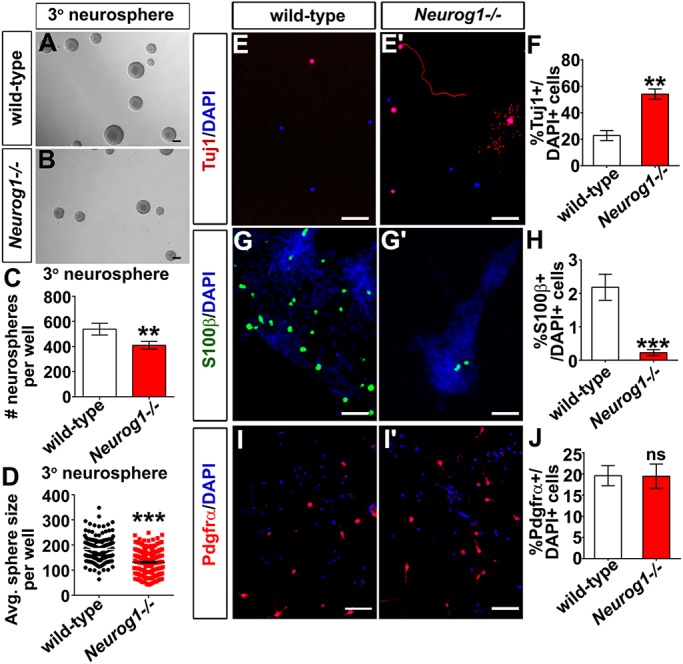
***Neurog1^−/−^* cortical progenitors have a reduced proliferative capacity and an enhanced neurogenic capacity *in vitro*.** (A-D) Tertiary (A,B) neurospheres generated from wild-type and *Neurog1^−/−^* cortical progenitors. Quantitation of 3° (C) neurosphere number per well (*N*=3, *n*=9, two-tailed *t*-test). Quantitation of average size of 3° (D) neurospheres (*N*=3, *n*=180 spheres for both genotypes). (E-J) Differentiation assay, staining for Tuj1 (E,E′), S100β (G,G′) and Pdgfrα (I,I′). Quantification of Tuj1^+^ (F), S100β^+^ (H) and Pdgfrα^+^ (J) cells from wild-type and *Neurog1^−/−^* progenitors (*N*=3, *n*=3, two-tailed *t*-test). Data are mean±s.e.m. in C,D,F,H,L. **P<0.01, ***P<0.001. Scale bars: 100 µm in A,B; 50 µm in E,E′,G,G′,I,I′.

To further investigate the developmental potential of the *Neurog1^−/−^* cortical progenitor pool, we used a neurosphere assay, which allows for the retrospective identification of self-renewing, proliferative neural stem cells (Pastrana et al., 2011). E12.5 wild-type and *Neurog1^−/−^* cortical cells were cultured in serum-free media containing basic fibroblast growth factor (bFGF) and epidermal growth factor (EGF), and neurosphere formation was assayed after 10 days in culture as a surrogate measure of stem cell number. E12.5 *Neurog1^−/−^* cortical cells formed significantly fewer primary (1°) neurospheres than wild type (decrease to 79.0%; Fig. S5A-C), and they were 69.0% smaller (Fig. S5D). These data are consistent with the reduced proliferative capacity of *Neurog1^−/−^* cortical progenitors *in vivo.* However, because 1° neurosphere cultures are a mix of different cell types, including progenitors, neurons and inhibitory niche signals, we also generated secondary (2°) (Fig. S5A′,B′) and tertiary (3°) (Fig. 4A,B) neurospheres by dissociating 1° or 2° spheres, respectively, and plating them at clonal density for 7 DIV (Pastrana et al., 2011). 2° spheres derived from *Neurog1^−/−^* neural cells were also reduced in number and size (73.6% fewer; 84.2% smaller; Fig. S5C′,D′), but as these are also an impure population, the more compelling data to assess neural stem cell function was the reduction to 76.1% in number of 3° spheres (Fig. 4C) that were also smaller in size decrease to 72.0%, Fig. 4D). *Neurog1* loss is thus associated with a decline in neural stem cell proliferation and self-renewal.

Another crucial feature of neural stem cells is multipotency, which is the ability to differentiate into three neural lineages: neurons, astrocytes and oligodendrocytes (Pastrana et al., 2011). To test multipotency, dissociated cortical progenitor cells were plated at low density in differentiation media lacking bFGF and EGF, and containing B27, a serum-free supplement that induces neurogenesis. Consistent with their increased ability to undergo neurogenesis *in vivo*, E12.5 *Neurog1^−/−^* cortical cells had a 2.37-fold increase in their ability to undergo neurogenesis compared with wild-type cortical cells *in vitro*, as assessed by the number of Tuj1^+^ neurons (Fig. 4E-F). In contrast, E12.5 *Neurog1^−/−^* cortical progenitors had a reduction to 10.3% in the differentiation of astrocytes, but these numbers were very small, likely because we assessed astrocytic potential outside of the normal developmental window (0.22-2.18% of all cells; Fig. 4G,G′,H). There were also no changes in the number of oligodendrocytes derived from E12.5 *Neurog1^−/−^* cortical progenitors (Fig. 4I-J). The most significant effect of the loss of *Neurog1* on early cortical progenitors is thus an increase in neuronal differentiation.

### *Neurog1* is required within the Neurog2^+^ cortical progenitor pool

**Fig. 5.**
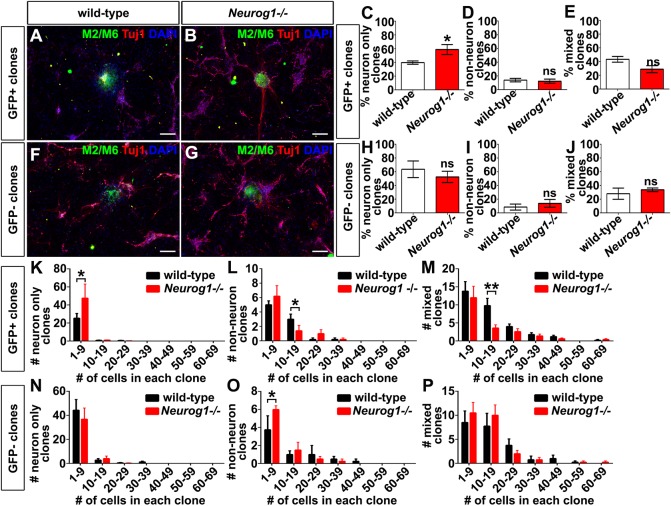
***Neurog1* is required in the Neurog2^+^ cortical progenitor pool.** (A-J) Clonal analysis of GFP^+^ (A-E) (*N*=5, clone number below) and GFP^−^ (F-J) (*N*=4, clone number below) clones. M2/M6 and Tuj1 immunostaining of *Neurog2^GFP^*^KI/−^ (‘wild-type’) (A,F) and *Neurog2^GFP^*^KI/+^; *Neurog1^−/−^* (B,G) clones derived from GFP^+^ (A,B) and GFP^−^ (F,G) cortical progenitors. Percentage of clones derived from GFP^+^ progenitors that are neuron only (wild type, 122; *Neurog1*^−/−^, 135; two-tailed *t-*test) (C), non-neuronal (wild-type, 36; *Neurog1*^−/−^, 41; two-tailed *t-*test) (D) and mixed (wild type, 133; *Neurog1*^−/−^, 90; two-tailed *t-*test) (E). Percentage of clones derived from GFP^−^ progenitors that are neuron-only clones (wild type, 195; *Neurog1*^−/−^, 164; two-tailed *t-*test) (H), non-neuronal clones (wild-type, 26; *Neurog1*^−/−^, 33; two-tailed *t-*test) (I) and mixed clones (wild-type, 88; *Neurog1*^−/−^, 95; two-tailed *t-*test) (J). (K-P) Size distribution of neuron only (K,N), non-neuronal (L,O) and mixed (M,P) clones derived from GFP^+^ (K-M) and GFP^−^ (N-P) E12.5 wild-type and *Neurog1*^−/−^ cortical progenitors (two-way ANOVA with Sidak's multiple comparison test). Data are mean±s.e.m. *P<0.05, **P<0.01. Scale bars: 50 µm.

As Neurog1 is expressed in only a subset of cortical progenitors (Fig. 1), the previous differentiation assay from dissociated cortical cells included both *Neurog1*-positive and -negative progenitors. To determine whether the changes in neurogenesis were specific to the proneural^+^ subset of progenitors, we took advantage of the high rate of *Neurog2* co-expression in *Neurog1*^+^ cells (Fig. 1R) and used FACS to isolate GFP^+^ and GFP^−^ cortical progenitors from *Neurog2^GFPKI/+^* embryos that were either wild type (*Neurog1^+/+^*) or *Neurog1*-null mutants (*Neurog1^−/−^*). To promote differentiation, progenitor cells were cultured at clonal density on a feeder layer of dissociated P2 rat cortical cells for 7 days (as described by Nieto et al., 2001). Clones derived from murine cortical progenitors were identified by immunostaining with M2/M6, which recognises only mouse cells and not rat cells (as described by Nieto et al., 2001). Mouse clones were then classified based on co-labelling with Tuj1, a neuronal marker, as either neuron-only, non-neuronal or mixed (Fig. 5A,B,F,G).

Within the GFP^+^ pool, *Neurog1*^−/−^,*Neurog2^GFP^*^KI/+^ progenitors gave rise to 1.48-fold more neuron-only clones compared with wild-type GFP^+^ progenitors (Fig. 5C). In contrast, there were no differences in the numbers of non-neuronal (Fig. 5D) and mixed identity (Fig. 5E) clones derived from GFP^+^, *Neurog1*^−/−^ cortical progenitors compared with wild type. Moreover, a requirement for *Neurog1* was not observed in the GFP^−^ populations, as there were no differences between wild-type and *Neurog1^−/−^* progenitors in their ability to give rise to neuron-only clones (Fig. 5H), non-neuronal clones (Fig. 5I) or mixed identity clones (Fig. 5J).

We also examined clone size as a readout of progenitor behaviour, revealing that neuron-only clones in the <9 cell range were the only ones expanded in the GFP^+^ pool of *Neurog1^−/−^* cortices (1.87-fold increase; Fig. 5K). There were also, respectively, reductions to 46.7% and 36.7% in the numbers of non-neuronal (Fig. 5L) and mixed (Fig. 5M) clones in the 10-19 cell range derived from GFP^+^ *Neurog1^−/−^* cortical progenitors. However, these changes do not reflect a difference in overall numbers of non-neuronal and mixed clones (Fig. 5D,E), but rather a reduced proliferative capacity of *Neurog1^−/−^* cortical progenitors. Finally, the size of neuronal (Fig. 5N), non-neuronal (Fig. 5O) and mixed (Fig. 5P) clones derived from GFP^−^ *Neurog1^−/−^* cortical progenitors was like wild-type controls, suggesting that *Neurog1* functions specifically within the Neurog2^+^ progenitor pool.

In summary, *Neurog1^−/−^* cortical progenitors have a reduced proliferative capacity and preferentially undergo neurogenesis. Moreover, the enhanced ability of *Neurog1^−/−^* progenitors to give rise to neurons is specific to the *Neurog2*-expressing progenitor population.

### *Neurog1* and *Neurog2* interfere with each other's ability to induce neurogenesis

**Fig. 6.**
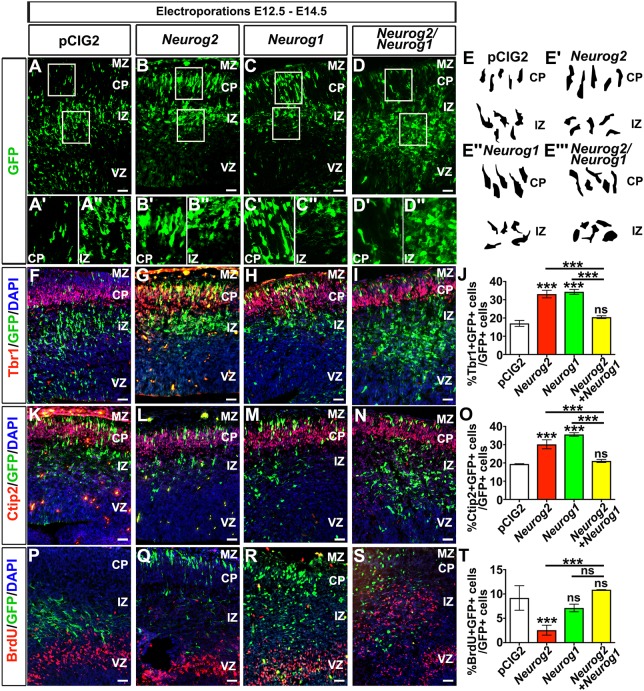
***Neurog1* and *Neurog2* are cross-repressive in cortical gain-of-function assays.** (A-T) E12.5 to E14.5 electroporations of pCIG2 (A,F,K,P), *Neurog2* (B,G,L,Q), *Neurog1* (C,H,M,R), or *Neurog2* and *Neurog1* (D,I,N,S). Insets are higher-magnification images of GFP^+^ cells in the cortical plate (A′-D′) and intermediate zone (A″-D″). Cell tracings showing cell morphologies in the CP and IZ of cortices electroporated with pCIG2 (E), *Neurog2* (E′), *Neurog1* (E″) or *Neurog2* and *Neurog1* (E‴). (F-I,K-N,P-S) Co-labelling of GFP with Tbr1 (F-I) and Ctip2 (K-N), and colocalisation with BrdU (P-S) in cortices electroporated with pCIG2 (F,K,P), *Neurog2* (G,L,Q), *Neurog1* (H,M,R), or *Neurog2* and *Neurog1* (I,N,S). (J,O,T) Quantitation of GFP^+^Tbr1^+^/GFP^+^ cells (J), GFP^+^Ctip2^+^/GFP^+^ cells (O) and GFP^+^BrdU^+^/GFP^+^ cells (T). *N*=3, *n*=9, one-way ANOVA with post-hoc Tukey for all counts. Data are mean±s.e.m. ***P<0.001. CP, cortical plate; IZ, intermediate zone; MZ, marginal zone; VZ, ventricular zone. Scale bars: 100 µm in A-D,F-I,K-N,P-S; 50 µm in A′,A″,B′,B″,C′,C″,D′,D″.

We hypothesised that *Neurog1* may repress neurogenesis within the Neurog2^+^ population by inhibiting *Neurog2* proneural activity*.* To test this model, we performed a gain-of-function assay by electroporating E12.5 cortical progenitors with pCIG2 expression constructs (that co-express GFP) for *Neurog2* or *Neurog1*. After 48 h, we compared the rate of neurogenesis with GFP-only transfected progenitors (pCIG2). Although most pCIG2 (Fig. 6A-A″) and *Neurog1* and *Neurog2* (Fig. 6D-D″) electroporated GFP^+^ cells remained in the IZ, more *Neurog2* (Fig. 6B-B″) and *Neurog1* (Fig. 6C-C″) transfected cells made it to the cortical plate, suggesting that they had undergone differentiation. Indeed, the GFP^+^ cells that made it to the cortical plate in *Neurog1* (Fig. 6C′,E″) and *Neurog2* (Fig. 6B′,E′) transfections had typical uni-polar or bipolar neuronal morphologies, whereas the GFP^+^ cells that remained in the intermediate zone (IZ) after pCIG2 (Fig. 6A″,E) and *Neurog1* and *Neurog2* transfections (Fig. 6D″,E‴) had a multipolar phenotype characteristic of newly differentiated neurons that initially stall in the upper SVZ/IZ before moving into the cortical plate (Noctor et al., 2004).

An assessment of Tbr1, a layer VI neuronal marker, revealed that misexpression of either *Neurog2* (1.94-fold increase; Fig. 6G,J) or *Neurog1* (2.01-fold increase; Fig. 6H,J) promoted the formation of supernumerary GFP^+^Tbr1^+^ neurons compared with pCIG2 alone (Fig. 6F,J), as previously reported for *Neurog2* (Dixit et al., 2014; Kovach et al., 2013; Mattar et al., 2008). In striking contrast, when *Neurog1* and *Neurog2* were electroporated together, the number of GFP^+^Tbr1^+^ neurons produced was reduced compared with when these constructs were electroporated alone (*Neurog1* and *Neurog2* versus *Neurog2*, decrease to 61.8%; *Neurog1* and *Neurog2* versus *Neurog1*, decrease to 59.6%; Fig. 6I,J). Instead, *Neurog1-Neurog2* co-electroporation produced a similar number of GFP^+^Tbr1^+^ neurons as in pCIG2 controls, consistent with the low number of GFP^+^ cells migrating out of the intermediate zone and into the cortical plate in both instances (Fig. 6I,J).

We next examined Ctip2, a layer V marker with increased in expression in *Neurog1^−/−^* cortices. In E12.5 to E14.5 electroporations, the number of Ctip2^+^GFP^+^ cells was increased by the misexpression of both *Neurog2* (1.55-fold increase) and *Neurog1* (1.83-fold increase) compared with pCIG2 controls (Fig. 6K-M,O). However, much like our observation with Tbr1, the number of Ctip2^+^GFP^+^ cells was reduced in *Neurog2*-*Neurog1* co-electroporated cortices when compared with *Neurog2* (decrease to 69.7%) and *Neurog1* (decrease to 59.2%) electroporated cortices (Fig. 6L-O). Compared with pCIG2 electroporated cortices, the number of Ctip2^+^GFP^+^ cells in *Neurog2*-*Neurog1* co-electroporated cortices did not significantly differ (Fig. 6K,N,O). Thus, although both *Neurog2* and *Neurog1* increase the differentiation of deep-layer Tbr1^+^ and Ctip2^+^ neurons, the co-expression of *Neurog2* and *Neurog1* interferes with their differentiation.

We observed large clusters of GFP^+^ cells in the IZ of pCIG2 (Fig. 6A,F) and *Neurog2*-*Neurog1* co-electroporated cortices (Fig. 6D,I), and we wondered whether these cells were proliferating or differentiated. To address this question, electroporated mice were injected with BrdU 30 min prior to sacrifice and cortical sections were co-stained for BrdU and GFP. Both pCIG2 (Fig. 6P) and *Neurog1* (Fig. 6R) electroporated cortices had similar numbers of BrdU^+^GFP^+^ proliferating cells, suggesting that *Neurog1* did not induce cell cycle exit. In contrast, *Neurog2* reduced the number of cortical progenitors incorporating BrdU to 30.7% compared with pCIG2 (Fig. 6P,Q,T). In contrast, co-expression of *Neurog2* with *Neurog1* prevented the induction of cell cycle exit by Neurog2, as assessed by the number of BrdU^+^GFP^+^ cells (Fig. 6S,T).

*Neurog1* and *Neurog2* thus both have proneural activity and induce the formation of deep layer neurons, but when they are co-expressed, they are cross-repressive, preventing neurogenesis from occurring. These data provide a model to explain why neurogenesis is transiently elevated in *Neurog1^−/−^* cortices, suggesting that the loss of the inhibitory activity of *Neurog1* allows *Neurog2* to initiate the process of neurogenesis more effectively.

### Neurog1 and Neurog2 proteins physically interact, and Neurog1 influences the expression of a select set of downstream transcriptional targets

**Fig. 7.**
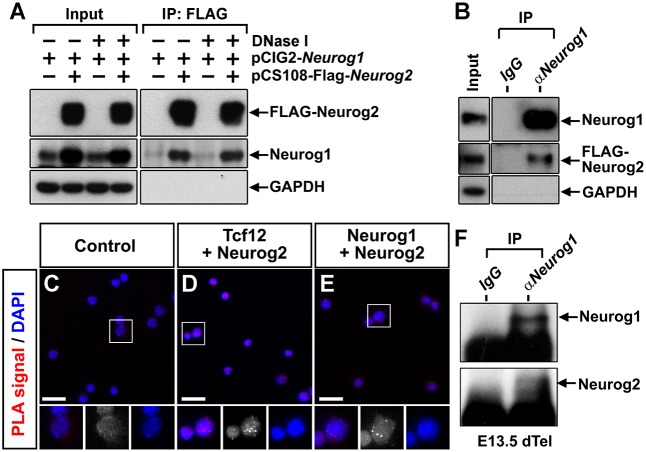
**Neurog1 and Neurog2 interact *in vitro* and *in vivo.*** (A,B) Co-immunoprecipitation of Neurog1 protein with FLAG-Neurog2 using anti-FLAG beads with or without DNaseI (A) or co-immunoprecipitation of FLAG-Neurog2 protein with Neurog1 using anti-Neurog1 antibodies (B) in 3T3 cells transfected with Neurog1 and FLAG-tagged Neurog2. (C-E) Proximity ligation assay (PLA) on dissociated E12.5 cortical progenitors, showing PLA signals in cells stained with no antibodies (negative control; C), Tcf12 and Neurog2 (D), and Neurog1 and Neurog2 (E). Areas outlined are shown at higher magnification underneath. (F) Co-immunoprecipitation of Neurog2 by Neurog1 antibodies from E13.5 cortical progenitors *in vivo*. In A,B,F, GAPDH is a loading control for input and a negative control for immunoprecipitation. Scale bars: 50 µm; 25 µm in higher magnifications.

From the above results, we speculated that one way in which Neurog1 and Neurog2 may negatively regulate each the activity of one another is through the formation of non-functional or alternatively functioning heterodimers (Bertrand et al., 2002). This explanation would be consistent with the specific requirement for *Neurog1* within the *Neurog2*^+^ progenitor pool. To assess whether Neurog1 and Neurog2 proteins physically interact, NIH-3T3 cells were transfected with *Neurog1* and Flag-tagged *Neurog2* expression plasmids. Anti-Flag immunoprecipitation followed by anti-Neurog1 western blotting revealed that Neurog2 could bring down Neurog1 protein (Fig. 7A). As both Neurog1 and Neurog2 interact with chromatin, if they bind to adjacent sites in the genome, they could be pulled down together by bridging DNA, rather than through a physical interaction. To test this possibility, we added DNaseI to the immunoprecipitate. Neurog1 was still brought down after DNaseI treatment (Fig. 7A, lanes 3 and 4 in After IP), suggesting Neurog1 and Neurog2 interact with each other in a chromatin-independent manner. A reciprocal experiment using an IgG control or anti-Neurog1 immunoprecipitation showed a specific pull-down of Neurog2 by Neurog1 (Fig. 7B). In this *in vitro* system, there is therefore a direct protein-protein interaction between Neurog1 and Neurog2 that is not mediated by DNA binding.

To further test Neurog1-Neurog2 protein interactions *in situ*, we set up a proximity ligation assay (PLA), which uses oligonucleotide-tagged secondary antibodies that can be ligated together when cognate proteins are in close proximity (<16 nm apart), allowing rolling circle amplification and detection with a fluorescently labelled probe (Bagchi et al., 2015). We performed PLA for Neurog1 and Neurog2 proteins, first in NIH-3T3 cells, which were transfected with pCIG2-*Neurog1* and pCIG2-*Neurog2*, which co-express GFP. Forty-eight h post-transfections, cells were immunostained with GFP (Fig. S6A-A‴), Neurog1 (Fig. S6B-B‴) or Neurog2 (Fig. S6C-C‴) alone, or Neurog1 and Neurog2 together (Fig. S6D-D″). Cells were then incubated with secondary antibodies conjugated to PLA probes, followed by DNA amplification of circularised DNA products that can be fluorescently labelled, and which are only generated when two proteins are close together, as a surrogate measure of physical interaction. Using this assay, a PLA signal was identified only when cells were co-immunolabelled with both Neurog1 and Neurog2 (Fig. S6D-D‴), verifying that these proteins interact *in vitro*.

To then assess *in vivo* interactions, we performed PLA experiments by using dissociated cortical cells with no primary antibody (negative control; Fig. 7C), antibodies to Neurog2 and Tcf12, a ubiquitous E-protein that is known to interact with Neurog2 (positive control; Fig. 7D), and antibodies to Neurog1 and Neurog2 (Fig. 7E). A PLA signal was visualised in cortical cells immunostained with both Neurog2-Tcf12 (Fig. 7D) and Neurog1-Neurog2 (Fig. 7E) but not in control wells (Fig. 7C), suggestive of protein-protein interactions *in vivo.* Finally, to provide direct support for Neurog1 and Neurog2 interactions *in vivo*, we immunoprecipitated Neurog1 from E13.5 cortical lysates, followed by an anti-Neurog2 western blot. We detected Neurog1 protein in the immunoprecipitate, and an enrichment of Neurog2 in only the Neurog1 pull-down lane (Fig. 7F). We therefore have evidence that Neurog1 and Neurog2 interact *in vitro* and *in vivo*, and our functional data suggest that this heterodimerisation likely reduces the proneural activities of these two transcription factors.

### Identification of *Neurog1-*regulated genes in the E12.5 neocortex

Finally, we addressed another non-mutually exclusive mechanism by which *Neurog1* might regulate the timing of neurogenesis, which is through the regulation of downstream gene expression. As *Neurog1* function has not been well studied in the neocortex, target genes are virtually unknown. We made the assumption that *Neurog1* and *Neurog2* may regulate similar targets, and focused on a set of genes that are known to be de-regulated in *Neurog2* loss- or gain-of-function assays, including genes involved in neural cell fate specification and neuronal differentiation (*Ascl1*, *Mef2c*, *Neurod2*, *Neurod4*/*Math3*, *Neurod6*/*Math2*, *Nhlh2*/*Nscl2* and *Fezf2*), and Notch signalling (*Dll1*, *Hes1* and *Hes5*) (Dennis et al., 2017; Kovach et al., 2013; Mattar et al., 2004, 2008).

**Fig. 8.**
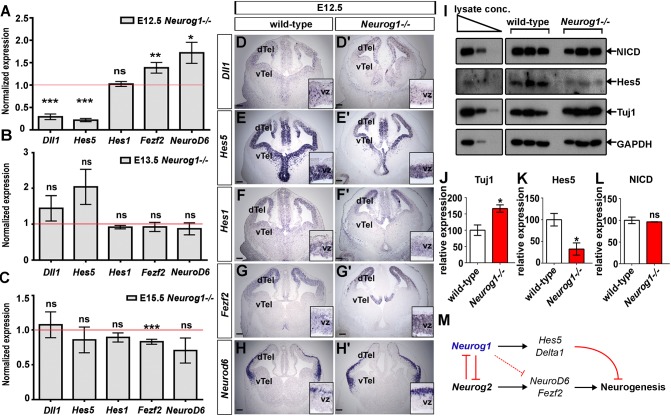
**Downstream effectors of *Neurog1* in early cortical progenitors.** (A-C) RTqPCR analysis of expression levels of potential *Neurog1*-regulated genes in E12.5 (A), E13.5 (B) and E15.5 (C) wild-type and *Neurog1^−/−^* cortices (*N*=3, *n*=9; comparing wild type with control for each gene using two-tailed *t*-tests). (D-H′) *In situ* hybridisation for *Dll1* (D,D′), *Hes5* (E,E′), *Hes1* (F,F′), *Fezf2* (G,G′) and *Neurod6* (H,H′) expression in wild-type and *Neurog1^−/−^* telencephalons at E12.5. Insets are high-magnification images. Scale bars: 100 µm (33 µm in insets). (I) Western blots for NICD, Hes5, Tuj1 and GAPDH proteins from E12.5 wild-type and *Neurog1^−/−^* cortices (three embryos for each genotype). (J) Densitometric measure of relative protein expression for Tuj1 (J), Hes5 (K) and NICD (L) in E12.5 wild-type and *Neurog1^−/−^* cortices (*N*=3, *n*=3; comparing wild type with control for each gene using two-tailed *t*-tests). (M) Schematic depicting non-mutually exclusive mechanisms by which *Neurog1* regulates neurogenesis. Data are mean±s.e.m. *P<0.05, **P<0.01 ***P<0.001. dTel, dorsal telencephalon; vTel, ventral telencephalon; vz, ventricular zone.

By RTqPCR, there were decreases to 31.3% and 26.7% in *Dll1* and *Hes5* expression, respectively, in E12.5 *Neurog1^−/−^* cortices, whereas *Hes1* levels were unaffected (Fig. 8A). These genes are all in the Notch pathway, which promotes progenitor cell proliferation and negatively regulates neuronal differentiation (Bansod et al., 2017; Zhang et al., 2018). RNA *in situ* hybridisation showed that *Dll1* (Fig. 8D,D′), *Hes5* (Fig. 8E,E′) and *Hes1* (Fig. 8F,F′) were expressed throughout the E12.5 telencephalic VZ, and *Hes5* and *Dll1* were at apparently lower levels in *Neurog1^−/−^* cortices. The downregulation of Notch signalling genes in E12.5 *Neurog1^−/−^* cortical progenitors is consistent with the reduced proliferative capacity of these cells. Moreover, in line with the enhanced neurogenesis of E12.5 *Neurog1^−/−^* cortical progenitors, there was an increase in the expression of neuronal differentiation genes, such as *Fezf2* (Shimizu et al., 2010) (1.38-fold increase) and *Neurod6* (Bormuth et al., 2013) (1.72-fold increase) (Fig. 8A). RNA *in situ* hybridisation showed that the expression of *Fezf2* (Fig. 8G,G′) and *Neurod6* (Fig. 8H,H′) was confined to the dorsal telencephalic VZ and CP, respectively, and both markers appeared to be upregulated in *Neurog1*^−/−^ cortices. However, by E13.5, the expression of all of these genes had normalised to wild-type levels when examined using RTqPCR (Fig. 8B) and RNA *in situ* hybridisation (Fig. S7A-E′), and by E15.5, *Fezf2* expression had even begun to decline (Fig. 8C, Fig. S7F-J′).

Finally, we asked whether we could detect a difference in Notch signalling in *Neurog1^−/−^* cortices using western blotting. We first confirmed that we could detect the enhanced neurogenesis in E12.5 *Neurog1^−/−^* cortices by western blotting with Tuj1, an early neuronal marker, revealing a 1.67-fold increase (Fig. 8I,J). We then examined Hes5 expression and revealed that this protein was reduced to 32.5% at the protein level in E12.5 *Neurog1^−/−^* cortices (Fig. 8I,K). However, this alteration in Hes5 expression levels was not due to an overall change in the Notch signalling pathway, as levels of the Notch intracellular domain (NICD), a marker of activated Notch signalling, were similar in E12.5 wild-type and *Neurog1^−/−^* cortices (Fig. 8I,L). Our data therefore suggest that *Neurog1* regulates Hes5 in a Notch-independent manner (Fig. 8M).

## DISCUSSION

Whether *Neurog1* functions as a bona fide proneural gene was initially called into question by the paradoxical finding of precocious neurogenesis in E12.5 *Neurog1*^−/−^ neocortices (Dixit et al., 2014; Schuurmans et al., 2004), contrasting with the typical neuronal loss observed in proneural mutants. By characterising *Neurog1* function in the developing neocortex in more detail, we found that, at early stages of neurogenesis, *Neurog1* acts as a competitive inhibitor of *Neurog2*, explaining in part why neurogenesis is transiently accelerated in *Neurog1* mutants*.* Specifically, we found that supernumerary deep-layer VI (Tbr1^+^) and V (Ctip2^+^) neurons are born in the E12.5 *Neurog1*^−/−^ preplate, but this effect is transient, as neuronal numbers are normalised by E15.5. Using clonal analysis, we demonstrated that the increase in neurogenesis in *Neurog1^−/−^* progenitors was specific to the *Neurog2*^+^ subset of cortical progenitors. Furthermore, we observed that although overexpression of *Neurog1* and *Neurog2* could each independently increase the number of deep-layer neurons, neurogenesis was diminished when *Neurog1* and *Neurog2* were co-electroporated. This cross-inhibition is likely mediated in part by the formation of Neurog1-Neurog2 heterodimers, which may either be non-functional or have alternative gene targets. In this model, the absence of *Neurog1* would create a mild *Neurog2* gain-of-function phenotype. A precedent has been set for a scenario in which binding partners influence proneural bHLH function, as Neurog2-Neurog2 homodimers function differently from Neurog2-E47 heterodimers (Li et al., 2012).

Another, non-mutually exclusive inhibitory mechanism likely involves the requirement for *Neurog1* to induce the expression of negative regulators of neurogenesis (*Dll1* and *Hes5*) and repress the expression of positive regulators (*Fezf2* and *Neurod6*) (Fig. 8M). *Hes5* is of interest as it appears to be repressed in a Notch-independent fashion, as levels of NICD are unchanged in *Neurog1^−/−^* cortices despite the downregulation of *Dll1. Hes5* expression may be directly upregulated by *Neurog1*, whereas the repressive effects on *Fezf2* are more likely to be indirect, as Neurog1 is a transcriptional activator. One possibility is that Neurog1 inhibits Neurog2 function, which is required to turn on *Fezf2*. Another possibility is that there are regulatory interactions between *Hes5* and *Fezf2*; indeed, *Fezf2* expression is suppressed when *Hes5* is overexpressed (Bansod et al., 2017). Conversely, Fezf2 protein can bind and repress the *Hes5* promoter (Shimizu et al., 2010), implying a cross-repressive relationship. Furthermore, in zebrafish, low *fezf2* expression has been linked to Notch signalling, with progenitors with low *fezf2* expression being more proliferative (Berberoglu et al., 2014). *Hes5* itself is also important for regulating temporal identity transitions in the developing neocortex. When *Hes5* is overexpressed in the cortex, there is precocious upper-layer neurogenesis and gliogenesis; conversely, deep-layer neurogenesis is prolonged in *Hes5* knockouts (Bansod et al., 2017). Mechanistically, Hes5 directly represses *Hmga1* and *Hmga2*, epigenetic factors that promote deep-layer neurogenesis (Bansod et al., 2017; Kishi et al., 2012). It will be of interest in the future to determine whether *Hmga1* and *Hmga2* levels are also altered in *Neurog1^−/−^* cortices, helping to explain the altered timing of cortical neurogenesis.

Distinct roles for *Neurog1* and *Neurog2* in the developing neocortex have also been observed in other contexts. For example, *Neurog2* and not *Neurog1* is sufficient to induce the expression of reelin (Dixit et al., 2014). Moreover, only *Neurog1* has been shown to be a negative regulator of astrogliogenesis, acting via the transcriptional induction of *miR-9*, which targets several LIF pathway genes (He et al., 2005; Zhao et al., 2015). In addition, Neurog1 competes for Smad and Stat effectors that operate downstream of BMP and cytokine signalling, respectively, to induce an astrocyte identity (Sun et al., 2001). Notably, the decline in *Neurog1* expression at E15.5, which is mediated by polycomb (PcG) proteins (Onoguchi et al., 2012), is in keeping with its role as an inhibitor of gliogenesis, as this is when astrocytes start to differentiate. In contrast, *Neurog2* continues to be expressed after E15.5, extending into the postnatal period. Thus, either *Neurog2* cannot inhibit astrocyte differentiation, or its activity is inhibited at later stages. Consistent with the latter possibility, *Neurog2* can only induce rapid neurogenesis before E14.5 (Li et al., 2012), possibly because there is a global compaction of cortical chromatin mediated by high mobility group A (HMGA) proteins at these later stages (Kishi et al., 2012), such that Neurog2 target genes are no longer accessible. In addition, at later developmental stages, Neurog2 is phosphorylated by GSK3, a proline-directed serine threonine kinase that alters co-factor binding, inhibiting its activity in the neocortex and changing target recognition in the spinal cord (Li et al., 2012; Ma et al., 2008).

A second confounding finding was the overall increase in preplate thickness in *Neurog1^−/−^* and *Neurog1^−/−^; Neurog2^−/−^* cortices that was not observed in *Neurog2^−/−^* mutants (Schuurmans et al., 2004). These data suggest that only *Neurog1* controls preplate thickness, and that this function is not dependent on *Neurog2*, which is contrary to other findings in this manuscript (i.e. the increased formation of neuron-only clones by *Neurog1^−/−^* cortical progenitors occurs only in *Neurog2^GFP+^* cells)*.* However, the expanded preplate in *Neurog1^−/−^; Neurog2^−/−^* cortices is populated by GABAergic neurons, whereas it is populated by glutamatergic (Tbr1^+^) neurons in *Neurog1^−/−^* mutants. The most likely possibility is that *Neurog1* normally inhibits Neurog2 function to reduce its ability to promote glutamatergic neurogenesis, but in the absence of *Neurog2*, *Ascl1* is upregulated and *Neurog1* is also required to limit its activity. A later normalisation of neuronal number is likely due in part to the return to normal levels of neurogenic and neuronal differentiation genes.

In summary, we have identified a non-canonical role for *Neurog1* as a negative regulator of neurogenesis, similar to its role as an inhibitor of astrogliogenesis (He et al., 2005; Sun et al., 2001; Zhao et al., 2015). Although the ability of *Neurog1* to regulate early cortical neurogenesis could be enacted through either Neurog2 dimerisation or downstream signalling, these models are by no means mutually exclusive, and could rather represent two parallel methods of regulating neurogenesis.

## MATERIALS AND METHODS

### Animals and genotyping

Animal care was approved by the University of Calgary and the Sunnybrook Research Institute Animal Care Committees in agreement with the Guidelines of the Canadian Council of Animal Care (CCAC). *Neurog1*^−/−^ (Schuurmans et al., 2004) and *Neurog2^GFPKI^* (Britz et al., 2006) transgenic mice were maintained on a CD1 background as previously reported. All tissue was obtained at embryonic stages indicated, and embryos were not separated based on sex. For timed pregnancies, the morning the vaginal plug was detected was designated embryonic day (E) 0.5. Genotyping was performed with the following PCR primers and conditions: *Neurog2^GFPKI^*, 35 cycles of 98°C for 1 s and 60°C for 30 s using primers for wild-type (Neurog2*F and Neurog2*R) and mutant (VD187 and ZF92) alleles; Neurog2*F, 5′TAGACGCAGTGACTTCTGTGACCG 3′; Neurog2*R, 5′ ACCTCCTCTTCCTCCTTCAACTCC 3′; VD187, 5′ GGACATTCCCGGACACACAC 3′; ZF92, 5′ GCATC ACCTTCACCCTCTCC 3′; *Neurog1* wild-type and mutant, 98°C for 30 s, 35 cycles of 98°C for 1 s and 58°C for 25 s, and 72°C for 1 min using primers for wild-type (Neurog1*F and Neurog1*R) and mutant (Neurog1*F and Neurog1MT*R) alleles; Neurog1*F, 5′-TCCAAACCTCCTGTCCGTCTG-3′; Neurog1*R, 5′-TTCCTGCTCTTCGTCCTGGG-3′; Neurog1MT*R, 5′-CGTGTCTTGT AGTTCCCGTCATC-3′.

### Tissue processing

Embryos were dissected at the stages indicated and fixed overnight at 4°C in 4% paraformaldehyde (PFA) in phosphate-buffered saline (PBS) (pH 7.5). Embryos were washed three times for 10 min in PBS, and then immersed in 20% sucrose/1×PBS overnight at 4°C. Embryos were then embedded in OCT compound and stored at −80°C. Sections (10 µm) were cut on a cryostat.

### BrdU labelling

We performed intraperitoneal injections of BrdU at 100 µg/g body weight at the specified times (i.e. 30 min or 24 h before dissection). For immunolabelling, sections were treated with 2 N HCl for 25 min at 37°C prior to immunostaining following the established protocol.

### Neurosphere assay

E12.5 cortices were dissected, and cells were dissociated in 0.125% trypsin (ThermoFisher Scientific #15090046) at 37°C for 8 min. Trypsin was inhibited using 20% FBS, and cells were collected at 520 ***g***, resuspended in 1 ml DMEM, seeded at 8000 cells/ml in 24-well plates (Coles-Takabe et al., 2008) and cultured for 10 days in neurosphere media [DMEM/F12 (3:1), human FGF2 (40 ng/ml), human EGF (20 ng/ml), B27 supplement minus vitamin A (2%), penicillin/streptomycin (0.1%), Fungizone (40 ng/ml), 1 μM cyclopamine]. After 10 days, primary neurospheres were counted and photographed using an AxioVision program (Carl Zeiss). For secondary and tertiary neurospheres, 1° or 2° neurospheres were dissociated with Accumax (Innovative Cell Technology, AM-105) for 15 min at 37°C, cultured and analysed as above.

### Differentiation assay

Five-thousand cells were dissociated as described above, plated in eight-well chamber slides coated with poly-L-ornithine and laminin, and incubated for 1 day in stem cell media, containing KnockOut D-MEM/F12, GlutaMax-I supplement [2 mM), bFGF (20 ng/ml), EGF (20 ng/ml), 2% StemPro Neural Supplement, penicillin/streptomycin (0.1%) and Fungizone (40 ng/ml)]. Media were then replaced by neuronal differentiation medium [Neurobasal medium, 2% B27 Serum-Free Supplement (ThermoFisher Scientific, 17504), GlutaMax-I supplement (2 mM)], Astrocyte differentiation medium [D-MEM, 1% N-2 Supplement (ThermoFisher Scientific, 17502), GlutaMax-I supplement (2 mM), 1% FBS) or oligodendrocyte differentiation medium [Neurobasal medium, 2% B-27 Serum-Free Supplement (ThermoFisher Scientific, 17504), GlutaMax-I supplement (2 mM), T3 (Sigma, cat. D6397)]. Media were replaced every 2 days for 10 DIV. Cells were fixed with 4% PFA for 15 min at room temperature and immunostained using mouse anti-Tuj1 antibody (neuronal III β-tubulin, 1/500, Covance, MMS-435P), goat anti-Pdgfrα antibody (1/500, R&D Systems, AF1062) or rabbit anti-S100b antibody (1/500, Dako, Z031129). Secondary antibodies were conjugated to Alexa fluor 568 (Molecular Probes) or Alexa fluor 488 (Molecular Probes).

### Clonal analysis

E12.5 cortices from *Neurog1*^+/−^; *Neurog2^GFP/+^* heterozygous intercrosses were dissociated using trypsin, FACS sorted into GFP^+^ and GFP^−^ populations and plated at 200 cells per well on a feeder layer of rat cortical cells in neurosphere media containing bFGF (2 ng/ml) for 7 days. Cells were stained using M2/M6 (DSHB) to distinguish mouse cells from rat cells. Clones were stained with anti-Tuj1 (neuronal III β-tubulin, 1/500, Covance, MMS-435P) and quantified as containing only neurons, no neurons or a mix of neurons and other cell types. Clone size was also assessed.

### Immunohistochemistry

All antibodies used in this study have been used previously, and the expression patterns were as expected and are referenced throughout the text. Cryosections (10 µm) were blocked in PBT (0.1% Triton-X100 in PBS) containing 10% horse serum for 1 h at room temperature. Sections were incubated in primary antibodies diluted in blocking solution overnight at 4°C as follows: rabbit anti-Tbr1 (1:800, Abcam, ab31940), rabbit anti-GFP (1:500, Molecular Probes, A-11122), goat-anti-GFP (1:1000, Abcam, ab5450), rabbit anti-Pax6 (1:500, Convance, PRB-278P), rabbit anti-Tbr2 (1:500, Abcam, ab23345), rabbit anti-phospho-histone H3 (pHH3; 1:500; Millipore Biotechnology, 06-570), rat anti-BrdU (1:20, Serotec, OBT0030S), mouse anti-NeuN (1:500, Chemicon, MAB377), rabbit anti caspase3 active (Ac-3, Abcam, ab2302), rat anti-Ctip2 (1:100, Abcam, ab18465), rabbit anti-Ki67 (1:200, Vector laboratories, VP-K451), mouse anti-Satb2 (1:350, Abcam, ab51502), goat anti-Neurog1 (1:200, Santa Cruz, sc-19231), rabbit anti-Neurog1 (a gift from Jane Johnson, UT Southwestern, Dallas, TX, USA; Gowan et al., 2001) and goat anti-Neurog2 (1:100, Abcam, 154293). Slides were washed three times in PBT and incubated for 1 h at room temperature in secondary antibodies conjugated to Alexa568 (1:500, Molecular Probes) or Alexa488 (1:500, Molecular Probes). Slides were washed three times in PBS, stained with DAPI (1/10,000 for 5 min), washed three times and mounted in Aquapolymount (Polysciences).

### RNA *in situ* hybridisation

RNA *in situ* hybridisation was performed as described previously (Alam et al., 2005; Touahri et al., 2015). Riboprobes were generated to *Neurog1*, *Neurog2*, *Neurod6*, *Hes1*, *Hes5* and *Dll1* as previously described (Cau et al., 2000; Dunwoodie et al., 1997; Fode et al., 2000; Gradwohl et al., 1996; Hirata et al., 2006).

### *In utero* electroporation

Surgeries were performed as previously described (Dixit et al., 2011; Mattar et al., 2008). DNA included a pCIG2 control vector expressing GFP alone and pCIG2-*Neurog1* and pCIG2-*Neurog2*, all at 3 μg/μl. DNA was injected into the lateral ventricles of E12.5 telencephalons using borosilicate needles and a Femtojet microinjector, and electroporation was performed with a BTX electroporator (7 pulses, 55 mV, 7 s interval). Animals recovered after surgery and pups were collected on E14.5.

### RT-qPCR

E12.5 dorsal telencephalons were dissected out and RNA was extracted with TRIzol according to the manufacturer's instructions (Thermo Fisher Scientific, 15596026). We collected cortices from three embryos from each genotype and performed three biological replicates. We extracted RNA and generated cDNA using a RT2 primer assay kit and following the instructions provided (Qiagen 330001). Qiagen RT^2^ qPCR primers included *Gapdh* (PPM02946E), *B2m* (PPM03562A), *Hrpt* (PPM03559F), *Ascl1* (PPM31367F), *Dll1* (PPM25198A), *Fezf2* (PPM28244A), *Hes1* (PPM05647A), *Hes5* (PPM31391A), *Mef2c* (PPM04548A), *Neurod2* (PPM25186A), *Neurod4* (PPM25613A), *Neurod6* (PPM25253A), *Nhlh1* (PPM24807A), and*Nhlh2* (PPM31392C). We used the delta-delta Ct method to calculate relative expression levels, using three housekeeping genes to normalise (*Gapdh*, *B2m* and *Hrpt*).

### Western blotting and immunoprecipitation

NIH-3T3 (ATCC CRL-1658) cells were transfected with pCIG2-*Neurog1* and pCS108-*Neurog2*-FLAG expression vectors using Lipofectamine 3000 reagent (Invitrogen, L3000015), according to the manufacturer's protocol. Forty-eight hours post-transfection, the cells were harvested and lysed in NET2 lysis buffer (0.05% NP40, 150 mM NaCl, 50 mM Tris-Cl, pH 7.4) with protease (1× protease inhibitor complete, 1 mM PMSF), proteasome (7.5 µM MG132) and phosphatase (50 mM NaF, 1 mM NaOV_3_) inhibitors. Lysate (400 µg) was immunoprecipitated with anti-FLAG M2 beads (Sigma) overnight at 4°C. Half the samples were incubated with DNaseI (2 U/ml; Ambion). FLAG beads were washed five times in lysis buffer, resuspended in SDS-PAGE loading dye and run on 10% SDS-PAGE gels for western blot analysis with goat-anti-Neurog1 (1:10,000, Santa Cruz). For *in vivo* immunoprecipitates, lysates were prepared from E13.5 cortical cells in NET2 lysis buffer as above, and 200 µg of protein (in 400 µl volume) was immunoprecipitated with 4 µg of normal goat IgG (Santa Cruz, sc-2028) or goat-anti-Neurog1 (Santa Cruz, sc-19231), incubated overnight at 4°C on a rocker with 100 µl Protein A/G PLUS agarose beads (Santa Cruz sc-2003), processed as above and run on 15% SDS-PAGE gel.

Western blots were performed as described previously (Li et al., 2012) with goat anti-Neurog1 (1:1000, Santa Cruz, sc-19231), rabbit anti-Neurog1 (1:1000, Abcam, ab66498), goat anti-Neurog2 (1:1000, Abcam, 154293), rabbit anti-FLAG (1:2000, Cell Signaling, #2368), rabbit anti-GAPDH (1:5000, Cell Signaling, 2118), rabbit anti-Notch (Cleaved) (NICD, 1:1000, Cell Signaling, 4147), rabbit anti-Hes5 (1:1000, Millipore, Ab5708) and mouse anti-Tuj1 (neuronal III β-tubulin, 1:1000, Covance, MMS-435P). Films were developed using an ECL kit (EG Healthcare) following the manufacturer's instructions.

### Proximity ligation assay

E12.5 cortices were dissociated in 0.125% trypsin for 15 min, resuspended in D-PBS and allowed to adhere to a poly-D-lysine- and laminin-coated chamber slide. The cells were fixed with 4% PFA for 15 min and washed twice with PBS. The PLA-Duolink probe protocol (Sigma-Aldrich) was followed as per the manufacturer's instructions. For the *in vitro* PLA assay, NIH-3T3 cells (ATCC CRL-1658; newly acquired from ATCC) were transfected with pCIG2 expression vectors as outlined (Li et al., 2012). Forty-eight hours post-transfection, cells were fixed with 4% PFA for 15 min and washed with PBS. The PLA-Duolink probe protocol (Sigma-Aldrich) was followed as per the manufacturer's instructions. Primary antibodies included rabbit anti-Neurog1 (1:500, a gift from Jane Johnson), goat anti-Neurog2 (1:200, Santa Cruz, sc-19233) and rabbit anti-Tcf12 (1:200, Proteintech Group, 14419-1-AP). A no-antibody negative control was also performed.

### Quantitation and statistics

Cell counts were performed on photomicrographs from three sections of the rostral neocortex (at the level of the lateral and medial ganglionic eminences in E12.5-E13.5 sections and at the level of the striatum in E15.5 sections). Experimental numbers and statistical tests for each experiment are described in the figure legends, and statistics were performed using Prism software (GraphPad). Biological replicates refer to the number of embryos or cell cultures analysed and are denoted as *N* values. The total number of technical replicates are referred to as *n* values. No samples were excluded from analysis.

## Supplementary Material

Supplementary information
