# Supplementary material for: A non-canonical role for the proneural gene Neurog1 as a negative regulator of neocortical neurogenesis
Source: Development. 2018 Oct 1;145(19):dev157719. doi: 10.1242/dev.157719 (PMC6198467; doi:10.1242/dev.157719)
Supplement: Supplementary information [file develop-145-157719-s1.pdf]

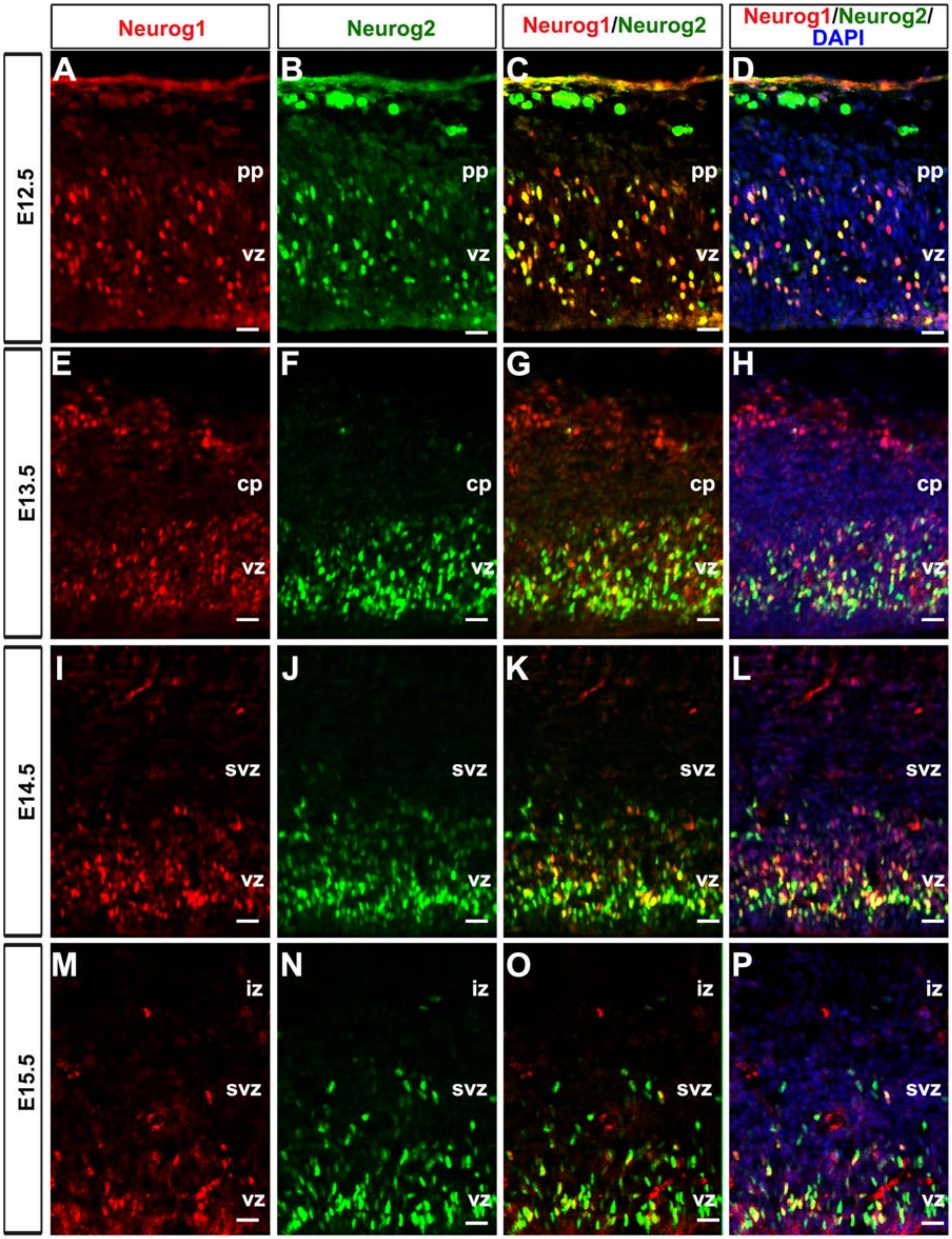

Figure S1. Co-expression of Neurog1 and Neurog2 in the developing neocortex.

Co-expression of Neurog1 (A,E,I,M), Neurog2 (B,F,J,N), and merged images without (C,G,K,O) and with (D,H,L,P) DAPI. CP, cortical plate; IZ, intermediate zone; SVZ, subventricular zone; VZ, ventricular zone. Scale bar = 50 µm.

Development • Supplementary information

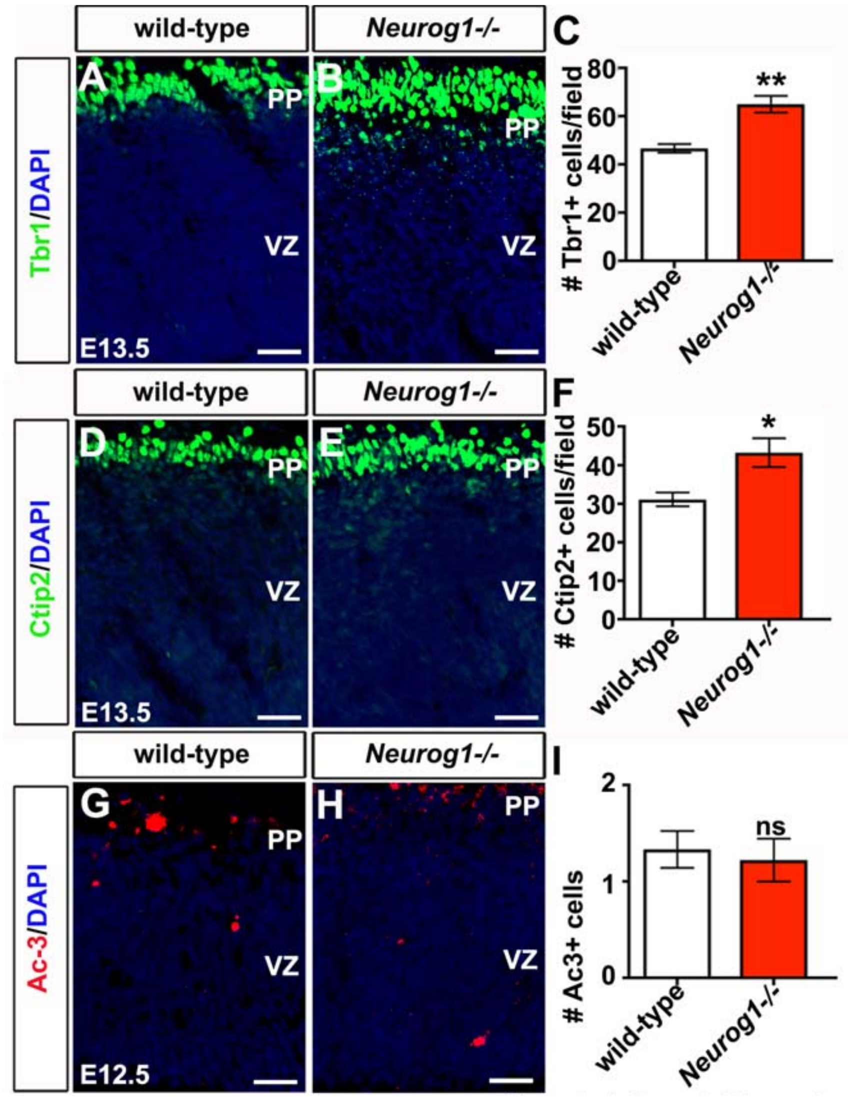

Figure S2. Increased deep-layer neurogenesis in E13.5 *Neurog1*$^{-/-}$ cortices.

Expression of Tbr1 (A,B), Ctip2 (D,E) and activated caspase 3 (Ac-3) (G,H) in E13.5 wild-type (A,D,G) and *Neurog1*$^{-/-}$ (B,E,H) cortices. Quantitation of Tbr1^{+} (C), Ctip2^{+} (F) and Ac-3+ (I) cells per field of view (N=3, n=9, unpaired *t*-test). Data are represented as mean ± s.e.m.. n = 3, p values are denoted as follows: $p < 0.05$ *, < 0.01 **, < 0.001***. PP, preplate; VZ, ventricular zone. Scale bars = 75 µm.

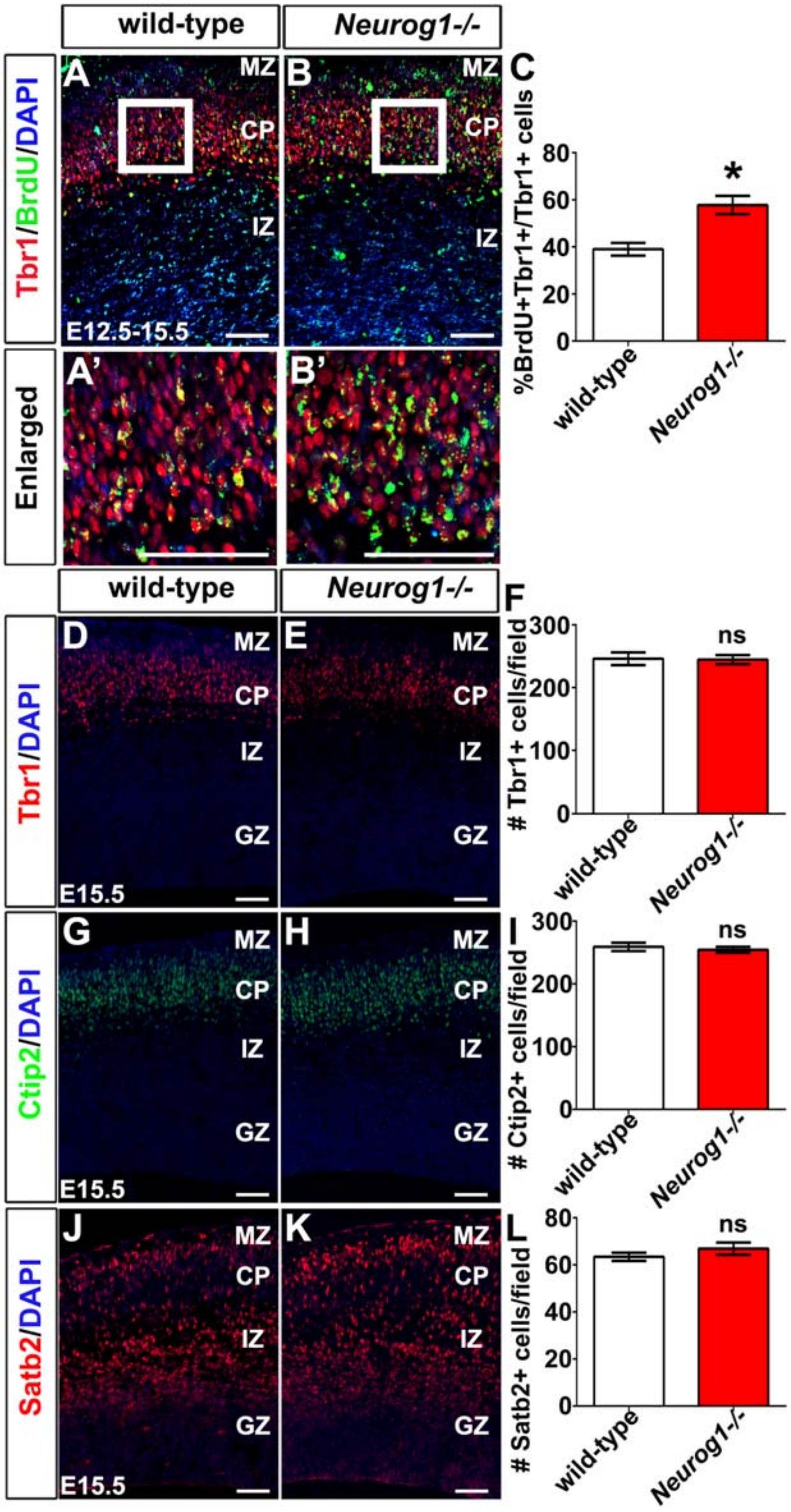

wild-type
Neurog1-/-
Tbr1/BrdU/DAPI
A
B
MZ
CP
IZ
E12.5-15.5
C
%BrdU+Tbr1+/Tbr1+ cells
*
80
60
40
20
0
wild-type
Neurog1-/-
Enlarged
A'
B'
D
E
F
Tbr1/DAPI
GZ
E15.5
Tbr1+ cells/field
300
200
100
ns
G
H
I
Ctip2/DAPI
Ctip2+ cells/field
J
K
L
Satb2/DAPI
Satb2+ cells/field

Figure S3. Neurogenesis is increased in early and not late embryonic *Neurog1*$^{-/-}$ cortices. (A-C) Co-localization of Tbr1 and BrdU in E13.5 wild-type (A,A') and *Neurog1-/-* (B,B') cortices after a 24 hr BrdU pulse. Quantitation of the co-localization of Tbr1 and BrdU (N=3, n=9; two-tailed t-test) (C). (D-L) Expression of Tbr1 (D,E), Ctip2 (G,H) and Satb2 (J,K) in E15.5 wild-type (D,G,J) and *Neurog1*$^{-/-}$ (E,H,K) cortices. Blue is DAPI counterstain. Quantitation of Tbr1^{+} (F), Ctip2^{+} (I), Satb2^{+} (L) cells per field of view (N=3, n=9; two-tailed t-test). Data are represented as mean ± s.e.m.. p values are denoted as follows: $p < 0.05$ *, < 0.01 **, < 0.001***. CP, cortical plate; IZ, intermediate zone; MZ, marginal zone; VZ, ventricular zone. Scale bars = 75 µm (A,A',B,B'), 100 µm (D,E,G,H,J,K).

Development • Supplementary information

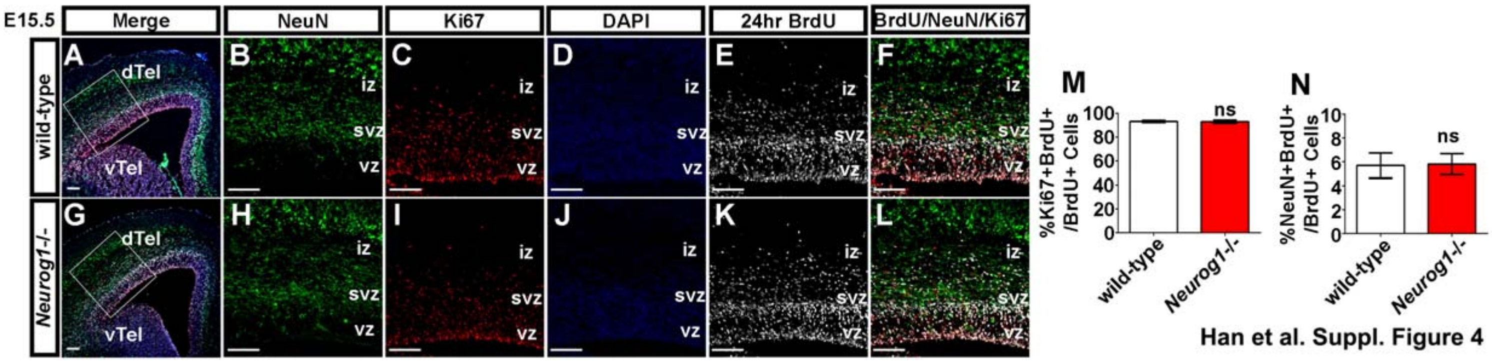

Figure S4. No differences in the p-fraction and q-fraction in E15.5 *Neurog1*$^{-/-}$cortices. Co-expression of NeuN (green, A,B,F,G,H,L), Ki67 (red, A,C,F,G,I,L) and co-localization of BrdU (white, A,E,F,G,K,L) and DAPI (blue, A,D,G,J) in E15.5 wild-type (A-F), and *Neurog1*$^{-/-}$ (G-L) cortices. (M) Quantitation of the leaving (q-) fraction, which is the proportion of BrdU-incorporating cells that co-localize with NeuN (N=3, n=9, two-tailed *t*-test). (N) Quantitation of the proliferative (p-) fraction, which is the proportion of BrdU-incorporating cells that co-localize with Ki67 (N=3, n=9, two-tailed *t*-test). Data are represented as mean ± s.e.m.. P values are denoted as follows: $P < 0.05$ *, < 0.01 **, < 0.001***. PP, preplate; VZ, ventricular zone. Scale bars = 100 µm.

Development • Supplementary information

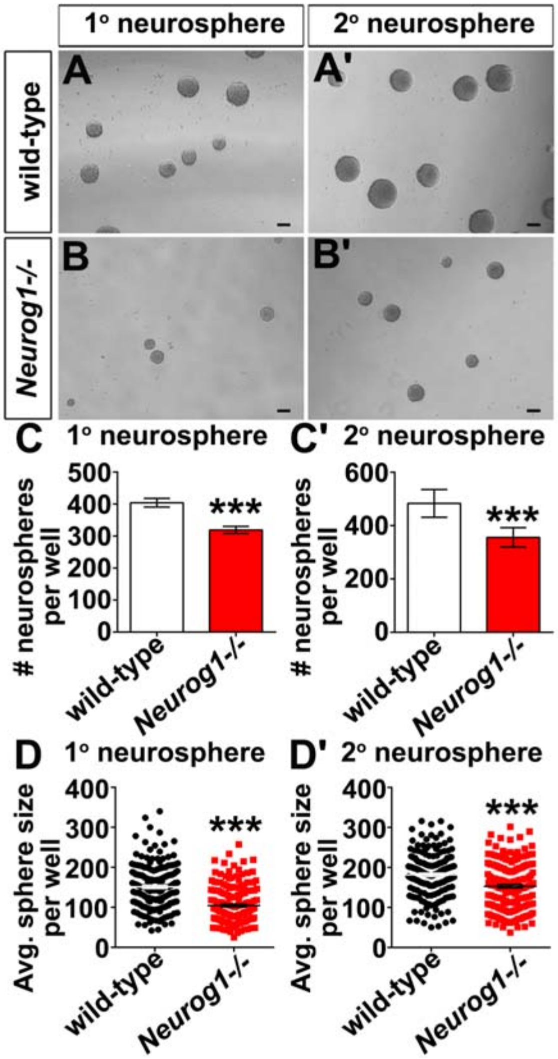

Figure S5. *Neurog1$^{-/-}$* cortical progenitors show reduced numbers and sizes of primary and secondary neurospheres. (A-D') Primary (A,B) and secondary (A',B') neurospheres generated from wild-type and *Neurog1$^{-/-}$* cortical progenitors. Quantitation of 1° (C) and 2° (C') neurosphere number per well (N=3, n=9, two-tailed t-test). Quantitation of average size of 1° (D) and 2° (D') neurospheres (N=3, n=180 spheres for both genotypes).

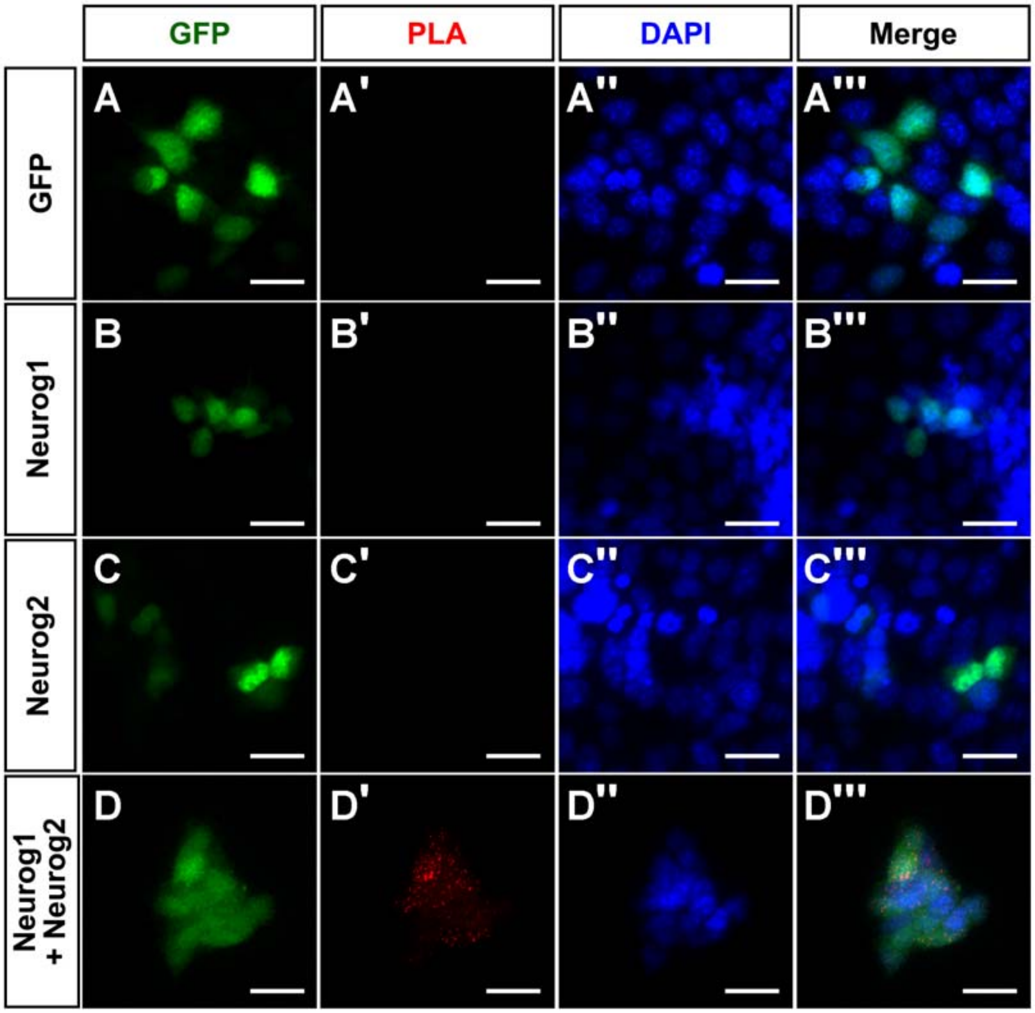

Figure S6. PLA assay to show Neurog1 and Neurog2 interactions *in vitro*. Immunostaining of NIH-3T3 cells 48 hr after co-transfection with pCIG2-*Neurog1* and pCIG2-*Neurog2*, using primary antibodies to GFP (A-A'''), Neurog1 (B-B'''), Neurog2 (C-C''') and Neurog1 and Neurog2 together (D-D'''), followed by PLA-conjugated secondary antibodies. GFP signal marks transfected cells (A-D), PLA signal marks cells with interacting proteins (A'-D'), DAPI marks all nuclei (A''-D'') and A'''-D''' are merged images. Scale bars = 25 µm.

Development • Supplementary information

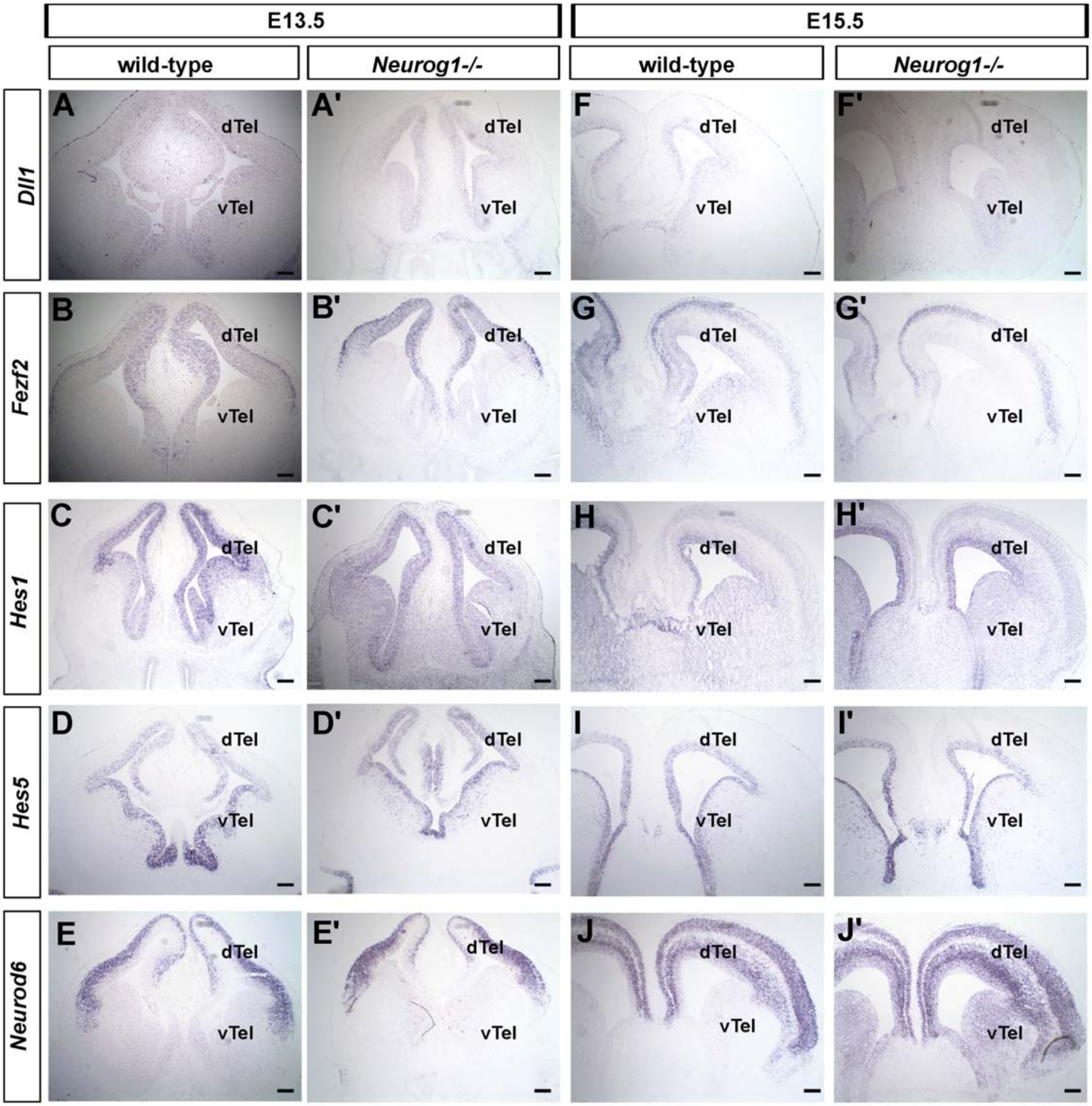

Figure S7. Expression of *Neurog1*-regulated genes at E13.5 and E15.5. (A-E) Expression of *Dll1* (A,A'), *Fezf2* (B,B'), *Hes1* (C,C'), *Hes5* (D,D') and *Neurod6* (E,E) in E13.5 wild-type (A-E) and *Neurog1*$^{-/-}$ (A'-E') telencephalons. (F-J) Expression of *Dll1* (F,F'), *Fezf2* (G,G'), *Hes1* (H,H'), *Hes5* (I,I') and *Neurod6* (J,J) in E13.5 wild-type (F-J) and *Neurog1*$^{-/-}$ (F'-J') telencephalons. dTel, dorsal telencephalon; vTel, ventral telencephalon. Scale bars= 100µm.

Development • Supplementary information
